# Oral Health Status and Oral Health Behaviour among 5- to 6-year-old Palestinian Schoolchildren – Towards Engagement of Parents and Schoolteachers for Oral Health through Schools

**DOI:** 10.3290/j.ohpd.b2448571

**Published:** 2021-12-18

**Authors:** Lamis Abuhaloob, Poul Erik Petersen

**Affiliations:** a Consultant, Ministry of Health, West Bank and Gaza Strip, State of Palestine. Idea, study concept, research design, field research, wrote the manuscript, statistical analysis.; b Professor, WHO Collaborating Centre for Community Oral Health Programmes and Research, University of Copenhagen, Denmark. Study concept, design, research instruments, wrote the manuscript, consulted on statistical analysis.

**Keywords:** dietary habits, oral hygiene behaviour, schoolchildren, school oral health promotion, socio-behavioural risk factors

## Abstract

**Purpose:**

1. To assess the oral health status and health behaviour of 5- to 6-year-oldchildren and the influence of socio-behavioural factors on oral health among children, and 2. evaluate dental knowledge and attitudes related to oral health promotion of children among mothers and schoolteachers in Palestine.

**Materials and Methods:**

This cross-sectional survey was carried out in 2017, and recruited samples of children (n = 3939, 53.8% boys, 44.8% girls) from 61 primary schools, their mothers and schoolteachers (n = 53). Participants were selected randomly by multistage stratified cluster sampling. Calibrated dentists performed oral examinations of 5- to 6-year-old schoolchildren based on WHO criteria. Mothers and teachers completed WHO self-administrated questionnaires to assess children’s oral health behaviour, as well as their knowledge and attitudes towards children’s dental care.

**Results:**

Caries prevalence of primary teeth was 83.4% and caries experience was high (dmf-s = 11.17). One-fourth of children suffered from pain or discomfort from teeth, and 57.7% of children had seen a dentist within the past 12 months, frequently due to pain or problems. Consumption of sugars was frequent and only 19.7% brushed their teeth every day. Neglect of dental visits, infrequent toothbrushing, being a child from families of urban settings and high socioeconomic status were greatly affected by caries. Mothers and schoolteachers had mostly positive attitudes towards school-based oral health care. However, the availability of dental health education materials was extremely low.

**Conclusion:**

The establishment of school-based oral health programmes, including effective use of toothpaste containing fluoride for caries prevention, is greatly needed to improve the oral health of Palestinian children. The introduction of fluoridated school milk is highly recommended. The establishement of school programmes should encompass active involvement of schoolteachers and mothers to promote the development of healthy lifestyles and sustainable oral health behaviours among schoolchildren. Provision of materials for health education by schoolteachers and mothers is urgently needed.

The World Health Organization (WHO) has encouraged countries to tackle the high burden of oral diseases in children through prevention and health promotion. In addition, the WHO stresses the adverse effect of poor oral health on general health, the quality of life, and the need for preventing the immense burden of oral diseases on children living in deprived areas.^12^

Long-term political instability and the severe social conflicts in the Palestinian territories have caused a serious deterioration of socioeconomic conditions, worsening of children’s quality of life and impairing psychosocial well-being of the Palestinian people.^6,19^ The United Nations Relief and Works Agency for Palestine Refugees in the Near East (UNRWA) as well as the Ministry of Health are facing financial crises as the result of cuts in funding by donors,^19^ and health resources for promoting people’s general health and oral health care have become severely limited.^9^

The poor oral health of Palestinian schoolchildren has been observed over the past two decades.^2^ Studies have shown that caries experience in primary teeth among young children of the West Bank was approximately twice as high as that of children living in the Gaza Strip. Among Palestinian children aged 12 and adolescents aged 16, the permanent teeth of around 40% of this group were affected by caries, and the majority of caries was left untreated. Moreover, the proportion of affected permanent teeth in need of dental care was 78% among 12-year-olds and 68% among the 16-year-olds. Finally, surveys documented that oral hygiene standards of children are rather poor and oral hygiene practice irregular.^2^

The most recent information about oral health in young children confirmed a high caries prevalence among 6-year-old schoolchildren. In 2016-2017, an average of 70.5% of Palestinian grade 1 schoolchildren suffered from caries in primary teeth. Only 6.4% of the children had teeth restored with fillings.^9^

Based on the surveillance data collected in Palestine,^2^ a comprehensive school health programme including oral disease prevention and health promotion has been established for primary school children (initially 5 to 6 years old). The programme is based on the WHO Health-Promoting Schools concept and focuses on development of healthy life styles, intervention against risk factors such as reduction of sugar consumption, promotion of a healthy diet low in sugars, ensuring good nutrition, effective use of fluoride, and improved oral hygiene of children. An expanded oral hygiene programme is integrated with general hygiene activities. The programme has been implemented in several representative primary schools in the Palestinian Territories. Schoolteachers engaged in this intervention have had comprehensive training in provision of classroom-based health education for schoolchildren and their parents, and they closely supervise the primary school children in regular toothbrushing with toothpaste containing fluoride.

Prior to the implementation of the national school oral health programme, information about oral health in children was collected among mothers and schoolteachers; such information was considered instrumental to planning and implementing school health education. The aim of the present baseline study was to assess the oral health behaviour of children and mothers, dental knowledge of mothers and teachers, and the oral health impact of socio-behavioural factors among children and mothers.

The specific objectives of the study were to: assess the burden of caries among 6-year-old schoolchildren in relation to gender, geographic location, urbanisation and socioeconomic status; evaluate schoolchildren’s oral health behaviour and dental-visit habits; evaluate the relative effect of socio-behavioural risk factors on caries experience of schoolchildren; judge the level of dental knowledge and attitudes towards children’s dental care among mothers and schoolteachers; and measure the involvement in health education by schoolteachers.

## Materials and Methods

A cross-sectional survey was conducted in 2017 including primary schools of Palestinian Territories. Within the West Bank and Gaza Strip governorates, a sample of 61 primary schools was selected randomly by multistage stratified cluster sampling. A total of 46 primary schools in the West Bank and 15 primary schools in the Gaza Strip participated. Schools of rural or urban areas were carefully chosen to ensure that the recruited 1st-grade schoolchildren would be balanced by gender, urbanisation and socio-economic status. All 1st-grade children aged 5 to 6 years and their parents were invited to contribute to the study; however, only very few fathers attended. The final sample of children and their mothers comprised 3939 participants (2567 children from the West Bank, 1372 children from the Gaza Strip); 53.8% were boys and 44.8% were girls. The response rate was 98.6% and all mothers signed an informed consent according to the Helsinki Committee guidelines. A total of 61 schoolteachers took part in the survey and the response rate was 86.9%.

### Data Collection

Approval by the Ministries of Health and Education was secured. The study proposal was prepared in collaboration with the WHO Collaborating Centre for Community Oral Health Programmes and Research, Denmark. Prior to clinical examination of children, mothers who participated in the study were asked to complete WHO self-administered questionnaires to measure the following variables:

- dental-visit habits of children and mothers
- services received by the child at the last dental visit
- mother’s knowledge about the causes and prevention of caries
- mother’s support/assistance in oral health care of children
- child’s self-assessment of caries
- child’s oral hygiene habits
- child’s dietary habits
- parents’ educational level
- parents’ work experience
- sex, age and region

In addition, the 1st-grade teachers completed a self-administered questionnaire for assessment of oral health in schoolchildren and engagement of teachers in school-based health education activities. Pre-tests took place prior to the survey to control validity and reliability of the questions. The original WHO questionnaires^24^ used for mothers and the questionnaire for schoolteachers were formulated in English, and then translated into Arabic.

### Clinical Assessments

At baseline, the Ministry of Health engaged 25 school dentists for the school project, and they examined the primary and permanent teeth of children for caries. The dentists were carefully trained in using the WHO criteria^24^ for recording caries at the cavity level. Calibration trials of examiners were undertaken prior to survey implementation. The participating dentists examined children at school, and the Kappa scores obtained were at least 0.9. During the survey, double examinations of approximately 10% of the children were performed to assess intra- and inter-examiner variability in the use of diagnostic criteria. The Kappa scores for intra- and inter-examiner consistency in the diagnosis of caries were 0.90 and 0.91, respectively.

### Data Analysis

The data were processed and analysed by means of the Statistical Package for the Social Sciences (SPSS PC version 20, IBM; Armonk, NY, USA). Bivariate and multivariate analyses of the data on oral health knowledge, attitudes and behaviour were based on frequency distributions. The prevalence rate of caries and the dmf-s (decayed, missing, filled primary tooth surfaces) and DMFS (decayed, missing and filled permanent tooth surfaces) values were calculated. Student’s t -test and ANOVA were used for the statistical evaluation of differences in caries means, while the statistical evaluation of proportions was performed using the chi-squared test. Consumption of sugary drinks and/or sweets was considered high if consumed ‘once or more a day’, moderate if consume ‘several times a week’ and low if consumed ‘sometimes or never’.

For assessment of the relative effect of socio-behavioural factors on caries experience, multiple dummy linear regression and logistic regression analyses were completed. In the logistic regression model, the dependent variable was represented by the dichotomous presence or absence of caries (i.e. dmf-s = 1 or more, or dmf-s = 0); the regression coefficient indicates the odds ratio (OR = P/l-P) of caries. The t-test was used for statistical evaluation of independent variables in the dummy regression, while the chi-squared test was used in the logistic regression analysis to estimate the OR.

## Results

### Caries Experience

The prevalence of caries in primary teeth was 83.4% and the mean number of tooth surfaces affected by caries (dmf-s) was 11.17 ± 11.29 (Table 1). On average, 11.4% of children were affected by caries in permanent teeth, and the mean caries experience (DMFS) was 0.26 ± 0.99. No difference in the caries prevalence rate in primary teeth was observed according to sex; however, boys (dmf-s = 11.56) had more surfaces affected by caries than did girls (dmf-s = 10.71). Further, 4.8% of boys and 14.6% of girls had caries in their permanent teeth, and the caries experience was higher among girls (0.34 ± 1.16) than among boys (0.19 ± 0.79) (p < 0.0001).

**Table 1:** Caries prevalence rate (%) and mean caries experience in deciduous teeth among Palestinian schoolchildren aged 5 to 6 years (dmf-s) according to geographic location and urbanisation

Indicator	Geographic location				Urbanisation			
	Gaza Strip	West Bank	Total	P-value	Urban	Rural	Total	p-value
	(n = 1372)	(n = 2567)	(n = 3939)		(n = 2210)	(n = 1729)	(n = 3939)	
Caries prevalence	81.7%	84.3%	83.4%	0.043	82.5%	84.4%	83.4%	0.111
d-s (M ± SD)	10.45 ± 10.77	9.21 ± 9.81	9.65 ± 10.17	<0.0001	9.69 ± 10.43	9.58 ± 9.84	9.65 ± 10.17	0.740
m-s (M ± SD)	1.64 ± 3.80	1.09 ± 3.19	1.28 ± 3.423	<0.0001	1.71 ± 3.94	0.74 ± 2.52	1.28 ± 3.42	<0.0001
f-s (M ± SD)	0.37 ± 1.41	0.18 ± 1.15	0.24 ± 1.24	<0.0001	0.27 ± 1.20	0.21 ± 1.30	0.24 ± 1.24	0.119
dmf-s (M ± SD)	12.45 ± 12.14	10.49 ± 10.75	11.17 ± 11.29	<0.0001	11.68 ± 11.74	10.53 ± 10.66	11.17 ± 11.29	0.002

M: mean value, SD: standard deviation.

In addition, Table 1 shows that the prevalence of caries in primary teeth was somewhat higher in children of West Bank (84.3%) than in the Gaza Strip (81.7%) (p < 0.05). In contrast, caries experience of children was considerably higher in the Gaza Strip (dmf-s = 12.45 ± 12.14) than in the West Bank (dmf-s = 10.49 ± 10.75) (p < 0.0001). The index components of decayed, missing and filled surfaces of primary teeth showed the same tendencies. Children living in rural areas were slightly more affected by caries in primary teeth (84.4%) compared to those living in urban areas (82.5%), but these differences were not statistically significant. Meanwhile, caries experience in primary teeth (dmf-s) (p < 0.01) and the ‘missing’ component (m-s) (p < 0.0001) were significantly higher among children living in urban than rural areas.

### Perceived Oral Health Problems and Dental Care

One-fourth of the children suffered from toothache or felt discomfort because of poor teeth, and one-fourth were not satisfied with the appearance of their teeth (Table 2). Most mothers (86.6%) stated that their children were in need of further oral hygiene instruction, 48.5% of the mothers claimed that their children required dental fillings, while tooth extraction was mentioned by 43.1% of mothers. Compared to the West Bank, in the Gaza Strip, a higher proportion of mothers reported that their child avoid smiling and laughing, missed school classes occasionally or for whole days because of toothache or discomfort of teeth, or that they were in need of dental fillings or tooth extraction.

**Table 2:** Percentages of Palestinian mothers who reported that their children aged 5 to 6 years had oral health problems and percentages of mothers claiming that children were in need for treatment

	Gaza Strip (n = 1372)	West Bank (n = 2567)	Total (n = 3939)
	%	%	%
Often experience toothache or feeling discomfort due to teeth	21.4	29.5	24.1
Avoid smiling and laughing because of teeth	19.9	11.4	14.5
Miss classes occasionally or for whole days because of toothache or discomfort of teeth	30.6	10.7	17.9
Not satisfied with appearance of child’s teeth	27.5	27.8	23.7
Need for instruction in proper oral hygiene	85.6	87.3	86.6
Need for dental fillings	58.5	41.9	48.5
Need for tooth extraction	44.4	42.6	43.1

Overall, 57.7% of the children had seen a dentist during the past 12 months and visits were related to pain or problems in 41.5% of the children (Table 3). At their last visit, three-quarters of the children received a dental examination, two-thirds received oral health instructions, and about one-fourth of the children received either tooth extraction (43.4%) or a filling (42.1%). Altogether, 58.6% of the mothers accompanied their children to the dentists. The proportions of children having visited a dentist and the reasons for visits during the past 12 months were very similar in Gaza Strip and West Bank. In the Gaza Strip, fillings, tooth extraction or gingival treatment for children were more common than in the West Bank.

**Table 3:** Percentage of Palestinian schoolchildren aged 5-6 years with certain dental-visit behaviours

	Gaza Strip (n = 1372)	West Bank (n = 2567)	Total (n = 3939)
	%	%	%
**Dental visits during the past 12 months**			
Once	24.5	24.1	24.3
Twice	15.3	13.5	14.2
Three times	8.3	6.6	7.2
Four times	3.8	3.3	3.4
More than four times	14.2	5.5	8.6
**Reason for last dental visit was pain/trouble with teeth or gums**			
Treatment at last dental visit	41.3	41.6	41.5
Provision of fillings	50.2	36.3	42.1
Removal of calculus	9.1	6.5	7.4
Tooth extraction	44.9	42.7	43.4
Examination of teeth	74.5	77.4	76.0
Treatment of gums	21.7	18.7	19.8
X-ray	6.9	13.0	10.7
Fluoride application	8.1	7.6	7.8
Instruction in care of teeth	63.2	67.0	65.1
**At last visit to the dentist, who went with the child?**			
Mother	60.4	57.1	58.6
Father	29.1	24.1	25.9
Both parents	0.0	2.8	1.7

### Oral Hygiene Habits and Sugar Consumption

One-fifth of the children brushed their teeth two or more times a day, while one-third of children cleaned them once a day. The use of fluoride toothpaste was reported for 60.4% of children (Table 4). In all, 43.9% of the children used wooden toothpicks and 25.5% used chewstick/miswaki to clean their teeth. In general, oral hygiene habits of children were stated more often in the West Bank than in the Gaza Strip.

**Table 4:** Percentage of Palestinian schoolchildren aged 5-6 years performing oral hygiene habits

	Gaza Strip (n = 1372)	West Bank (n = 2567)	Total (n = 3939)
	%	%	%
**How often do you brush your teeth?**			
Once a day	27.4	35.5	32.6
2 or more times a day	15.1	22.4	19.7
**Use of toothpaste containing fluoride**	53.5	64.1	60.4
**Use of tooth cleaning aids**			
Wooden toothpicks	37.6	47.5	43.9
Plastic toothpicks	5.3	10.4	8.5
Thread (dental floss)	9.0	10.9	10.1
Charcoal	4.6	4.0	4.2
Chewstick/miswak	25.7	25.7	25.7

Children often consumed sugary foods and drinks on a daily basis (Table 5). A high frequency of sugar consumption among children was particularly reported as biscuits, cakes, cream cakes, sweet pies, buns etc. (57.7%), sweets/candy (55.1%), and tea with sugar (52.0%). Compared to the West Bank, children of the Gaza Strip consumed sugar more often, e.g. as biscuits, cakes, cream cakes, sweet pies, buns etc, and chewing gum containing sugar. In addition, children in the Gaza Strip consumed milk or tea with sugar more often than did children of the West Bank. In contrast, the intake of soft drinks and sweets/candy was more frequent in children of the West Bank.

**Table 5:** Percentages of 5- to 6-year-old Palestinian schoolchildren consuming sugary foods and drinks at specific frequencies

Foods and drinks containing sugars	Several times a week			Every day			Several times a day			n = 3939
	Gaza Strip	West Bank	Total	Gaza Strip	West Bank	Total	Gaza Strip	West Bank	Total	
Biscuits, cakes, cream cakes, sweet pies, buns etc.	25.0	22.7	23.8	40.0	24.5	41.4	13.5	17.8	16.3	3163
Lemonade, cola or other soft drinks	33.9	30.1	31.5	13.0	27.3	22.1	4.1	8.6	7.1	3144
Jam/honey	9.6	15.1	13.1	5.5	7.4	6.7	2.0	1.9	2.0	3098
Chewing gum containing sugar	26.0	28.6	27.7	21.1	16.3	17.9	7.8	6.1	6.7	3055
Sweets/candy	21.9	23.5	22.9	36.2	40.0	38.6	16.0	16.7	16.5	3072
Milk with sugar	18.5	23.3	21.5	36.8	31.7	33.7	10.1	6.0	7.6	3075
Tea with sugar	15.2	20.9	18.8	46.5	36.0	39.9	15.9	9.7	12.1	3117

### Socio-behavioural Risk Factors and Caries of Schoolchildren

Table 6 shows the results from the multivariate analysis of socio-behavioural risk factors on caries experience in primary teeth (dmf-s) of children. Other factors being equal, sex, socioeconomic group, dental visits, and frequency of toothbrushing had significant effect on caries experience. Brushing teeth 2 or more times a day statistically significantly reduced the risk of having caries compared to children who never brushed their teeth. Rare visits to a dentist increased the risk of having caries by about five times. Children living in urban areas had higher level of caries experience than children living in rural areas, while the risk of disease (OR) was moderate. A substantial proportion of the fathers (71.4%) and mothers (67.9%) of the schoolchildren had not finished tertiary school. Meanwhile, children of fathers with a high education had relatively more caries.

**Table 6:** Multivariate analysis of caries experience (dmf-s) in Palestinian schoolchildren aged 5 to 6 years (n = 3939)

Independent variable	Category	dmf-s (β) (p-value)	OR (p-value)
Gender	Male	0.85 (0.018)	1.031 (0.734)
	Female	-	-
Urbanisation	Urban areas	1.14 (0.002)	0.896 (0.223)
	Rural areas	-	-
Dental visits	Often	1.338 (0.004)	1.234 (0.066)
	Occasionally	3.880 (0.0001)	2.452 (0.0001)
	Rarely	4.813 (0.0001)	3.884 (0.0001)
	Never	-	-
Brushing	Never	-	-
	Several (2-3) times a month	0.782 (0.294)	0.860 (0.406)
	Once a week	0.792 (0.193)	1.385 (0.054)
	Several (2-6) times a week	0.297 (0.671)	0.963 (0.829)
	Once a day	-0.190 (0.693)	0.791 (0.046)
	2 or more times a day	-1.622 (0.004)	0.772 (0.054)
Consumption of sugary drinks	High	0.380 (0.343)	1.055 (0.585)
	Moderate	-2.314 (0.169)	0.543 (0.076)
	Low	-	-
Consumption of sweets	High	0.401 (0.329)	1.035 (0.733
	Moderate	0.696 (0.871)	1.112 (0.922)
	Low	-	-
Mother’s educational level	High	-0.043 (0.923)	0.956 (0.682)
	Intermediate	-0.061 (0.892)	0.900 (0.333)
	Low	-	-
Father’s educational level	High	0.898 (0.057)	0.652 (0.949)
	Intermediate	0.063 (0.889)	0.320 (0.897)
	Low	-	-
R^2^		0.05	

Dependent variables in the linear regression are dmf-s (regression coefficient β), while in the logistic regression, odds ratio (OR) is calculated from presence /absence of caries. dmf-s ≥ 1 or more was considered as presence of caries, while dmf-s = 0 represented absence of caries.

### Level of Dental Knowledge and Attitudes of Mothers and Schoolteachers

The majority of mothers agreed with the statements concerning tooth decay and gum disease, the importance of toothbrushing, the role of parents in providing help to children in toothbrushing, restricting consumption of sugars, and the relevance of dental visits (Table 7). Meanwhile, one-fifth of mothers held the opinion that false teeth are less bother than natural teeth, and two-thirds of respondents stated that they were afraid of visiting a dentist because of possible pain. Seven in ten mothers agreed that using fluoride is a good way of preventing tooth decay. A relatively high proportion of mothers in the Gaza Strip were afraid of visiting dentists because of possible pain (71.1%), whereas no remarkable variations between the Gaza Strip and the West Bank were found for other attitude items.

**Table 7:** Percentage of mothers who agreed with different attitude items related to oral health

Items	Gaza Strip (n = 1372) %	West Bank (n = 2567) %	Total (n = 3939) %
Tooth decay can make me look bad	91.1	92.0	91.7
Keeping natural teeth is not that important	4.8	5.0	4.9
False teeth will be less of a bother than natural teeth	20.7	19.5	20.0
I’m afraid of going to the dentist because of possible pain	71.1	63.1	65.9
Regular visits to the dentist keep away dental problems	94.4	93.3	93.7
Brushing my teeth can prevent tooth decay	97.5	97.6	97.6
Brushing my teeth will keep me from having trouble with my gums	93.3	93.5	93.4
Eating and drinking sweet things does not cause tooth decay	15.8	11.3	12.9
Using fluoride is a good way of preventing tooth decay	72.2	72.6	72.4
Children less than 10 years old need help from adults in toothcleaning	83.7	84.9	84.1
Parents should restrict children’s consumption of sweets and sweetened drinks	90.8	89.3	90.4

Moreover, communication with parents (72.3%) was the leading source of knowledge about care of oral health in children. while teachers (28.7%), dentists (28.7%), and media (TV, radio) (14.0%) played a modest role (Table 8). The answers were similar for mothers in the Gaza Strip and the West Bank, except for a notably higher proportion of mothers in the West Bank (30.5%) who responded that teachers contribute to their oral health knowledge, as compared to the Gaza Strip (25.4%).

**Table 8:** Percentage of mothers reporting sources of knowledge on oral health important for 5- to 6-year-old schoolchildren

Key sources of information and media for oral health	Gaza Strip (n = 1372) %	West Bank (n = 2567) %	Total (n = 3939) %
Parents	72.9	71.9	72.3
Relatives	12.5	15.8	14.6
Teachers	25.4	30.5	28.7
TV, radio	12.5	14.8	14.0
Cinema	0.4	0.7	0.6
Newspapers/magazines	1.2	1.6	1.5
Dentist	28.9	28.6	28.7
Dental assistant	5.2	4.9	5.0
Medical doctor	0.8	1.6	1.3
Medical nurse	1.8	2.1	2.0
Video	3.9	3.9	3.9
Others	3.9	3.9	3.9

The attitudes of schoolteachers towards prevention of oral disease in children are shown in Table 9. Most teachers confirmed the statements about causes and prevention of oral disease, although knowledge about prevention of bleeding gums (75.5%) and the preventive effect of fluoride (81.1%) was somewhat lower. The vast majority of schoolteachers held the opinion that children under 10 years old need help from adults in toothcleaning. Furthermore, nearly eight in ten schoolteachers declared that they informed children about the importance of oral health during the school year. However, only 3.8% of the teachers stated that they had sufficient material for teaching children about oral health. The differences in answers by schoolteachers concerning working toward oral health at school were minor.

**Table 9:** Percentage of schoolteachers who agreed with certain attitude items related to working toward oral health in schools (n = 53)

	Gaza Strip (n = 19) %	West Bank (n = 34) %	Total (n = 53) %
Fluoride protects the teeth against decay	89.5	78.8	81.1
Toothbrushing prevents tooth decay	94.7	90.9	92.5
Toothbrushing prevents bleeding from gums	73.7	75.8	75.5
Improper consumption of sugar causes teeth to decay	94.7	93.9	94.3
Children less than 10 years old need help from adults in toothcleaning	89.5	93.9	92.5
Schoolteachers should teach children about diet and sugar	100.0	97	98.1
Schoolteachers should teach children about causes of decay and bleeding gums	94.7	97.0	96.2
Schoolteachers should teach children about fluoride and caries	94.7	87.9	90.6
Schoolteachers should teach children how to take care of their teeth	100.0	97.0	98.1
Have you told the children from your class about teeth and oral health during this school year?	73.7	78.8	77.4
Do you have sufficient material for teaching about oral health?	5.3	3.2	3.8

## Discussion

This survey was undertaken to assess the oral health situation of schoolchildren enrolled in a newly established national school oral health promotion programme in Palestine. This was the first survey of child oral health carried out in Palestine using the WHO Oral Health Survey Methods and standard examination criteria.^24^ In addition, the study intended to measure the oral health behaviour of schoolchildren, the level of dental knowledge and attitudes of mothers and schoolteachers. Furthermore, the baseline information was instrumental for the planning of school activities for oral health.

The prevalence of caries and the mean caries experience of primary teeth in Palestinian 5- to 6-year-olds were remarkably high; the findings may be considered a peak prevalence in Palestine since the scholastic year of 1998-1999.^2^ The caries figures were far higher than the WHO global goals for oral health by the years 2000 and 2020.^5^ Moreover, the study indicated that young schoolchildren in Palestine had a higher prevalence of Early Childhood Caries (ECC)^23^ than their counterparts in similar low-income countries. On the other hand, the caries prevalence was lower than that of the Philippines (90%).^4^

Since 1999, the Ministry of Health has published reports on oral health of 5- to 6-year-olds.^2^ The current report revealed that the caries prevalence in primary teeth among schoolchildren in the Gaza Strip (36.1%) was half that of children of the same age group in the West Bank (65.5%). These statistically significant differences in caries prevalence were attributed to high fluoride concentration in groundwater in the Gaza Strip (0.00–9.6 ppm F^-^) and very low fluoride concentration in groundwater (0.10–0.53 ppm F^-^) in the West Bank.^2^ In the current survey, however, the caries prevalence in the Gaza strip has grown closer to the prevalence values of children in the West Bank. This outcome may relate to the observation that children in the Gaza Strip tend to drink more water free of fluoride over time.^1^

According to the WHO,^11^ untreated disease is a dominant component of the caries burden among children of low- and middle-income countries; this finding reflects the limited dental health care coverage.^15^ In the current study of Palestinian 5- to 6-year-old schoolchildren, the rate of untreated caries (d-s) was 86% of the total caries experience in primary teeth (dmf-s), while the ‘filled’ component comprised only 1.7%. Caries experience in Palestine was statistically significantly higher among children living in urban areas compared to those living in rural areas. This is in line with similar results among children in urban areas in countries of Africa, Asia and the Middle East.^7,11,13^

### Self-assessment of Oral Health in Children

Consistent with the clinical observation of extremely high caries prevalence, high proportions of mothers reported that their child: suffered from toothache or they felt discomfort on the account of their teeth; often missed class because of symptoms from their teeth; or was dissatisfied with the appearance of her/his teeth. Moreover, substantial proportions of mothers stated that their child was in need of restorative care, tooth extraction or instruction in care of the teeth. In all, these findings indicated major health concerns, but they also confirmed the serious implications of poor oral health for the quality of life of the children.

### Dental Care

Approximately 60% of all schoolchildren visited a dentist over the past 12 months; typically, their mother accompanied them. The study revealed that a substantial number of dental visits were prompted by symptoms such as pain or problems with teeth or gums; however, tooth extraction or provision of fillings were the predominant reasons for visiting a dentist. It is worth noting that while two-thirds of the children received instructions in toothbrushing, the application of fluoride for prevention of caries was sporadic. Such a treatment pattern is concordant with similar surveys of children carried out in the Arab region,^3,14^ and bears very little resemblance to the preventive approaches offered to children in most Western countries during dental visits.^21,22^

### Oral Hygiene Habits and Consumption of Sugars

The poor oral hygiene practices documented here for children in Palestine are similar to results obtained in Saudi Arabia,^3^ Jordan,^18^ and countries of Africa.^16,20^ This study demonstrated a high need for improving oral hygiene habits of Palestinian schoolchildren. Only one-fifth of the children brushed their teeth at least twice a day, while one-third cleaned their teeth once a day. Consequently, it is must be emphasised that a substantial number of children did not use fluoride toothpaste. However, traditional means of cleaning the teeth such as the use of wooden toothpicks or chew sticks were used as alternatives.

The present study documented a high intake of sugary foods and drinks among children, which poses a severe risk of caries along with an elevated risk of obesity and poor nutrition. According to the mothers, children most frequently consumed biscuits, cakes, cream cakes, sweet pies, buns, or sweets and candy. Further, the children often had ‘hidden’ sugar on a daily basis, e.g. tea or milk with sugar, particularly among children in the Gaza Strip. High consumption of sugars is an important global issue. Recently, the WHO emphasised the health hazards of sugars, and the ‘WHO Guideline on Sugar Intake for Children and Adults’^10^ includes a strong recommendation to reduce daily intake of free sugars to less than 10% of total energy intake. A further reduction to below 5% of total energy intake would protect oral health throughout the course of life. The WHO advocates a number of strategies for public health intervention against the intake of sugars;^8^ for children, the health-promoting schools provide a unique setting for promoting oral health through a healthy diet.

### Socio-behavioural Risk Factors for Caries of Schoolchildren

International studies have shown that childhood caries is strongly affected by socio-behavioural factors.^17^ Such effects were also documented for Palestine; the absence of regular dental visits, infrequent toothbrushing, and high consumption of sweets and sugary drinks denoted a severe risk of caries. The multivariate analysis of caries experience in primary teeth (dmf-s) confirmed that neglecting dental visits, not brushing teeth at least twice a day, a high level of education of father, and being a child of a family living in urban areas statistically significantly increased the risk of caries. According to a study carried out in China,^13^ a higher risk of caries for children living in urban settings and with a high socioeconomic status was explained by a greater ability to buy and consume sugary drinks and foods.

### Dental Knowledge and Attitudes of Mothers and Schoolteachers

Unlike the situation in Kuwait and China,^13,14^ in Palestine, parents themselves were the primary source of knowledge about care of teeth, while media (TV, radio) played a limited role. Remarkably, the current study indicated a high awareness of prevention of caries and gingival disease for both key groups. Fortunately, the majority of mothers and schoolteachers confirmed the awareness of regular toothbrushing, use of fluoridated toothpaste, the relevance of adult assistance to toothcleaning of children less than 10 years old, and the importance of restricting the consumption of sugars for prevention of caries of children. However, it appears that extra efforts are required to improve the understanding of the role of fluoride in prevention of tooth decay. These results are very similar to what was reported in Tanzania/Zanzibar^16^ and China.^13^ Lastly, the positive oral health knowledge and attitudes of schoolteachers were apparently put into good practice, as three-fourths of them stated that they gave lessons about dental care. However, sufficient materials for teaching about oral health were available to only a very small minortiy of teachers.

## Conclusion

The present study has revealed that oral health conditions of schoolchildren in Palestine are not under control. Oral diseases and the unmet need for dental care cause much suffering and reduce the quality of life of Palestinian children. In general, this unfortunate situation is understandable, given that oral health problems may not be considered of primary interest for people living in areas of war, conflict, social instability and continuous political unrest. The prevalence of caries is higher than the WHO goals for children by the year 2020, and the unmet need for dental care is substantial. Very high consumption of sugars, poor oral health habits, little ability to buy toothpaste and toothbrushes because of poverty, limited access to dental care, lack of financially fair dental treatment, and the absence of preventive care are important explanatory factors. Establishing a formal school oral health programme based on the WHO Health-Promoting Schools concept is vital to the development of oral health and healthy lifestyles of Palestinian schoolchildren. The introduction of fluoridated school milk for prevention of caries is highly recommended. The firm engagement of parents and schoolteachers is considered essential for better oral health of schoolchildren. The current study provides valuable baseline information, which is instrumental to the development of health promotion activities and the organisation of population-directed oral disease prevention. The results of the study will be used in future evaluation of the newly established national school health programme in Palestine.
